# Supplementary material for: Quantifying and reducing spurious alignments for the analysis of ultra-short ancient DNA sequences
Source: BMC Biol. 2018 Oct 25;16:121. doi: 10.1186/s12915-018-0581-9 (PMC6202837; doi:10.1186/s12915-018-0581-9)
Supplement: Supplementary file 1 — Table S1. Summary of bacterial sequences. Table S2. Summary of S. cerevisiae sequences. Table S3. Summary of A. laibachii sequences. Table S4. Percentage of sequences with a given number of mismatches for each type of alignments. Table S5. Sequence length cutoffs in hominin ancient DNA studies [39–65]. Table S6. Modern human contamination. Figure S1. Characteristics of spurious and true alignments. Figure S2. Length distribution of mapped sequences. Figure S3. Proportion of spurious alignments by sequence length for SH samples. Figure S4. Spurious alignments in SH samples and the effect of different filters. Figure S5. Significance of Neandertal-lineage assignment for different minimum length cutoffs. Figure S6. Lineage assignment as a function of sequence length. (PDF 767 kb) [file 12915_2018_581_MOESM1_ESM.pdf]

Supplementary Information:
Quantifying and reducing spurious alignments for the analysis of ultra-short ancient DNA sequences

Cesare de Filippo[1,*], Matthias Meyer[1], Kay Prüfer[1,*]

1 Max Planck Institute for Evolutionary Anthropology, Leipzig, Germany

* Corresponding e-mail: cesare_filippo(at)eva.mpg.de, pruefer(at)eva.mpg.de

Table S1. Summary of bacterial sequences.

Length	All unique seq.	Mapped (%)	Classified spurious (%)	Spurious expected (%) ¶
20	143,050,589	91.362	98.10	96.67
21	143,305,294	76.659	98.18	96.83
22	143,476,993	96.892	97.59	95.45
23	143,633,334	89.772	97.63	95.65
24	143,767,397	73.932	97.50	95.83
25	143,881,911	52.541	97.35	96.00
26	143,993,048	32.013	97.29	96.15
27	144,098,076	16.993	97.28	96.30
28	144,191,391	8.126	97.26	96.43
29	144,289,373	3.598	97.23	96.55
30	144,376,099	1.515	97.16	96.67
31	144,458,818	0.622	97.09	96.77
32	144,535,683	0.255	96.90	96.88
33	144,614,643	0.107	96.76	96.97
34	144,682,942	0.047	96.38	97.06
35	144,754,552	0.023	95.57	97.14
36	144,815,313	0.012	93.78	97.22
37	144,882,597	0.007	92.16	97.30
38	144,947,978	0.005	87.05	97.37
39	145,005,407	0.003	82.10	97.44
40	145,061,336	0.003	70.83	97.50
total	3,029,822,774	25.798	97.57	96.06

¶ The expected proportion of spurious alignment assuming that all sequences have the maximum number of mismatches was calculated as 1-($M/3$) where $M = m/l$ (see Materials and Methods), that is the maximum proportion of mismatches allowed in the alignment (m) at a given length (l). This is divided by 3 because three are all alternative non-reference alleles one of which is the hg19 reference. Using the 'ancient' parameters for alignment, 2 and 3 maximum mismatches are allowed for sequences of length 20-21 bp and 22-41 bp, respectively.

Table S2. Summary of S. cerevisiae sequences.

Length	All unique seq.	Mapped (%)	Classified spurious (%)	Spurious expected (%) ¶
20	459,775	97.256	97.52	96.67
21	459,707	92.031	98.07	96.83
22	459,977	98.445	97.31	95.45
23	459,995	97.035	97.26	95.65
24	459,923	90.983	97.41	95.83
25	460,129	76.067	97.33	96.00
26	460,119	52.633	97.14	96.15
27	460,133	29.795	97.25	96.30
28	460,172	14.895	97.33	96.43
29	460,180	7.040	97.48	96.55
30	460,196	3.459	97.47	96.67
31	460,366	1.855	97.20	96.77
32	460,274	1.112	95.79	96.88
33	460,383	0.770	96.73	96.97
34	460,360	0.564	97.56	97.06
35	460,401	0.428	91.11	97.14
36	460,351	0.357	91.18	97.22
37	460,473	0.286	97.67	97.30
38	460,584	0.231	100.00	97.37
39	460,510	0.208	94.44	97.44
40	460,584	0.160	90.00	97.50
total	9,664,592	31.677	97.35	96.09

¶ Calculated as in Table S1.

Table S3. Summary of A. laibachii sequences.

Length	All unique seq.	Mapped (%)	Classified spurious (%)	Spurious expected (%) ¶
20	1,521,949	94.512	98.11	96.67
21	1,522,376	83.767	98.27	96.83
22	1,523,090	97.805	97.68	95.45
23	1,523,586	93.818	97.82	95.65
24	1,524,095	81.770	97.60	95.83
25	1,524,523	59.721	97.43	96.00
26	1,525,067	34.574	97.50	96.15
27	1,525,174	15.917	97.43	96.30
28	1,525,389	6.304	97.54	96.43
29	1,526,095	2.319	97.23	96.55
30	1,525,809	0.840	97.19	96.67
31	1,526,272	0.325	97.09	96.77
32	1,526,196	0.132	96.25	96.88
33	1,526,868	0.071	93.89	96.97
34	1,526,717	0.041	91.07	97.06
35	1,527,161	0.028	72.73	97.14
36	1,527,097	0.022	66.67	97.22
37	1,527,294	0.017	66.67	97.30
38	1,527,272	0.016	44.44	97.37
39	1,527,335	0.012	NA	NA
40	1,527,486	0.012	100.00	97.50
total	32,036,851	27.200	97.68	96.05

¶ Calculated as in Table S1.

Table S4. Percentage of sequences with a given number of mismatches for each type of alignments.

Alignment type	Sample	Number of mismatches			
		0	1	2	3
True	Mezmaskaya 1	0.00%	57.21%	30.03%	12.76%
True	Modern human (negative control)	0.07%	89.39%	8.80%	1.74%
Spurious	Mezmaskaya 1	0.25%	3.40%	24.48%	71.87%
Spurious	Bacteria (positive control)	0.12%	4.81%	30.05%	65.02%

Table S5. Sequence length cutoffs in hominin ancient DNA studies.

Length (bp)	References
25	Malaspinas2014 [39], Raghavan2014 [40], Martiniano2016 [41]
25,30¶	Raghavan2015 [42]
30	Green2010 [16], Rasmussen2011 [43] Gamba2014 [44], Olalde2014 [45], Allentoft2015 [4], Gallego-Llorente2015 [46], Jones2015 [47], Olalde2015 [48], Rasmussen2015 [49], Hofmanova2016 [50]
34	Cassidy2016 [51]
35	Meyer2012 [6], Fu2014 [52], Lazaridis2014 [53], Pruefer2014 [20], Skoglund2014 [54], Fu2015 [55], Guenther2015 [56], Meyer2016 [8], Pruefer2017 [21], Schlebusch2017 [57], Skoglund2017 [58], Hajdinjak2018 [59]
Not specified	Rasmussen2010 [60], Keller2012 [61], Rasmussen2014 [62], Seguin-Orlando2014 [63], Schiffels2016 [64], Sikora2017 [65]

¶ A 25 bp length cutoff was applied to one of the two samples because a 30 bp cutoff would have reduced drastically the data for this sample. Only sequences without indels were considered.

Table S6. Modern human contamination. We used positions where three archaic high-coverage genomes (Denisova, Altai and Vindija33.19) are all homozygous for the ancestral allele and at least 90% of modern humans carry the derived allele(s). The estimates of contamination are reported with binomial 95% confidence intervals calculated using the number of contaminants (Nc) and the total number of sequences (Nt).

	35bp			$L_{1\%}$			$L_{10\%}$		
SAMPLES	Estimate	Nc	Nt	Estimate	Nc	Nt	Estimate	Nc	Nt
SH Incisor	13.0% (2.8-33.6%)	3	23	13.0% (2.8-33.6%)	3	23	24.1% (13.5-37.7%)	13	54
SH Femur frag.	15.8% (3.4-39.6%)	3	19	13.0% (2.8-33.6%)	3	23	17.1% (6.6-33.6%)	6	35
SH Molar	0.0% (0.0-97.5%)	0	1	0.0% (0.0-97.5%)	0	1	33.3% (0.8-90.6%)	1	3
SH FemurXIII	50.0% (6.8-93.2%)	2	4	NA¶	0	0	50.0% (6.8-93.2%)	2	4
Mezmaiskaya1	2.6% (1.9-3.3%)	59	2305	3.30% (2.7-4.0%)	101	3041	3.5% (2.9-4.7%)	108	3048

¶ No sequence that overlapped informative positions was longer than the cutoff $L_{1\%}$ = 46 bp for SH FemurXIII.

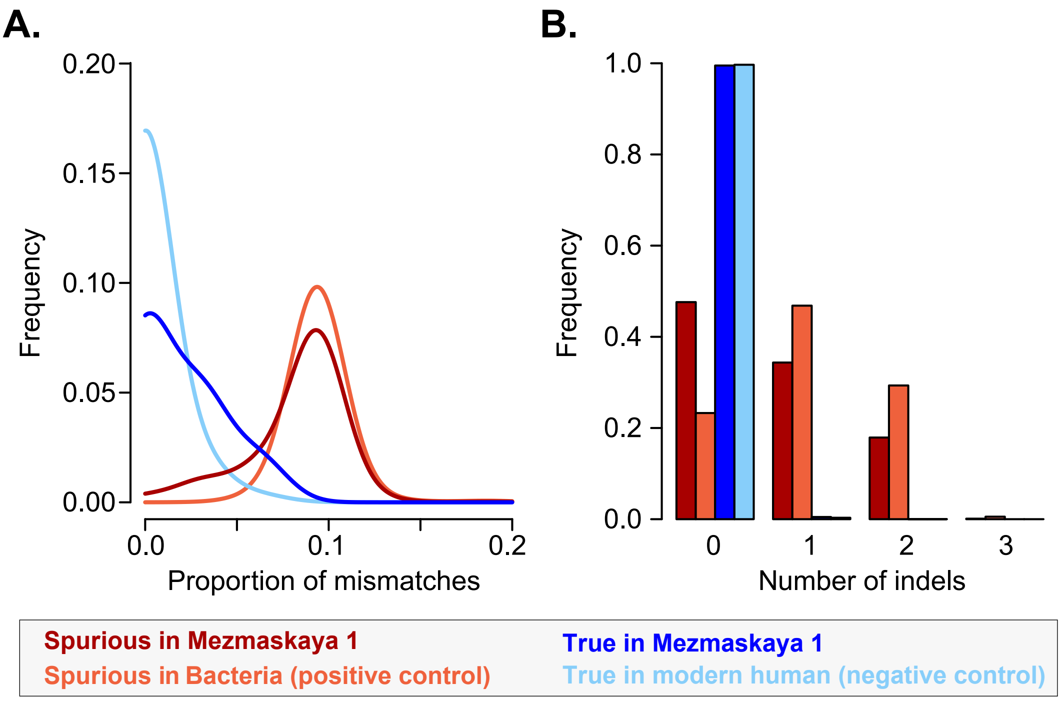

Figure S1. Characteristics of spurious and true alignments. Distributions of the proportion of mismatches (**A**) and distributions of the number of indels (**B**) for the spurious and true alignments detected in Mezmaskaya 1 (the same as in Figure 1C-D) and for the spurious and true alignments coming from the bacterial and the modern humans used as controls. The proportion of mismatches found in Mezmaskaya 1 (darkblue) are on average higher than in the modern human control (light blue) and this is due to the C-to-T changes as result of deamination in ancient samples. In Table S4 is reported the percentage of sequences having a given number of mismatches for each type of alignments.

.

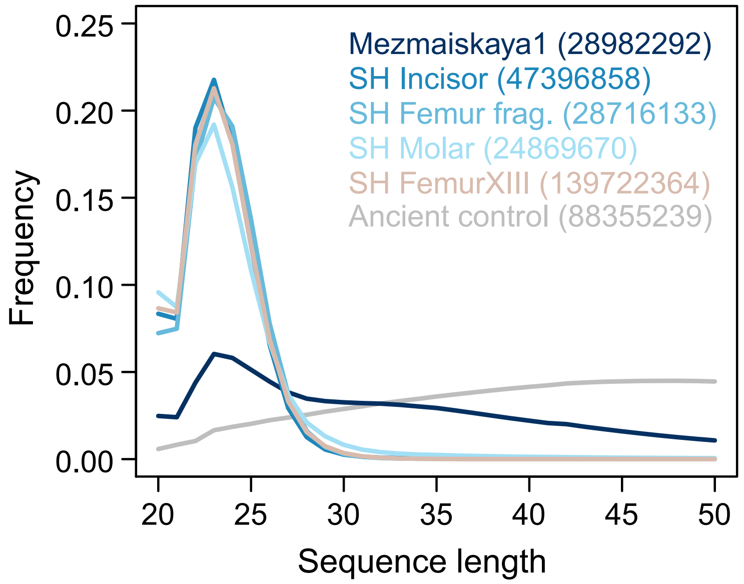

Figure S2. Length distributions of mapped sequences. The number in parentheses in the legend gives the total number of sapete come la penso.unique sequences of at least 20bp length. 57.1%, 99.5%, 99.7%, 97.0% and 99.2% sequences were shorter than 35 bp for Mezmaiskaya 1, SH Incisor, SH Femur fragment, SH Molar and SH Femur XIII, respectively.

.

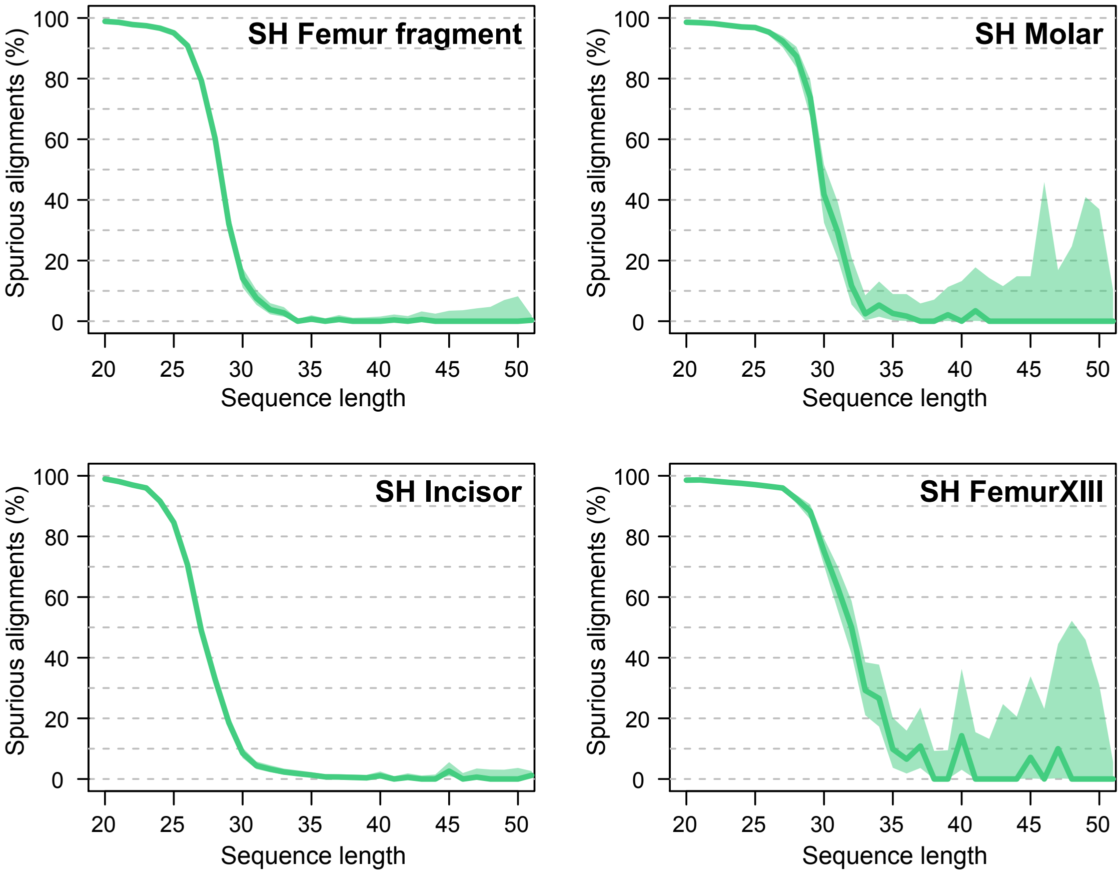

Figure S3. Proportion of spurious alignments by sequence length for SH samples. Proportion of spurious alignments by sequence length for SH samples. The filter "deam+indel" (see main text and Fig.2) was applied to all data. The line thickness shows the binomial 95% confidence interval. The point and confidence intervals at 51bp length show the estimates for sequences >50bp.

.

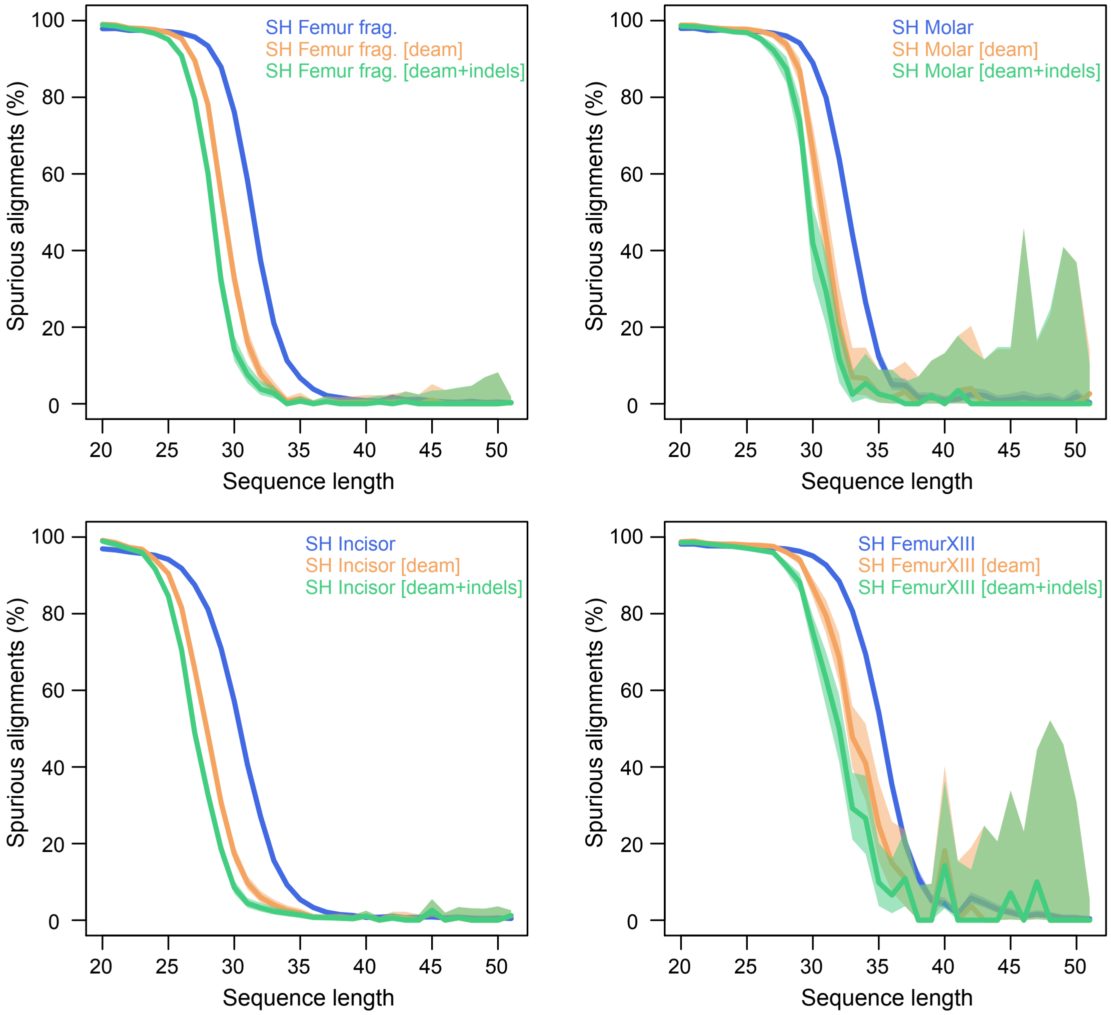

Figure S4. Spurious alignments in SH samples and the effect of different filters.

.

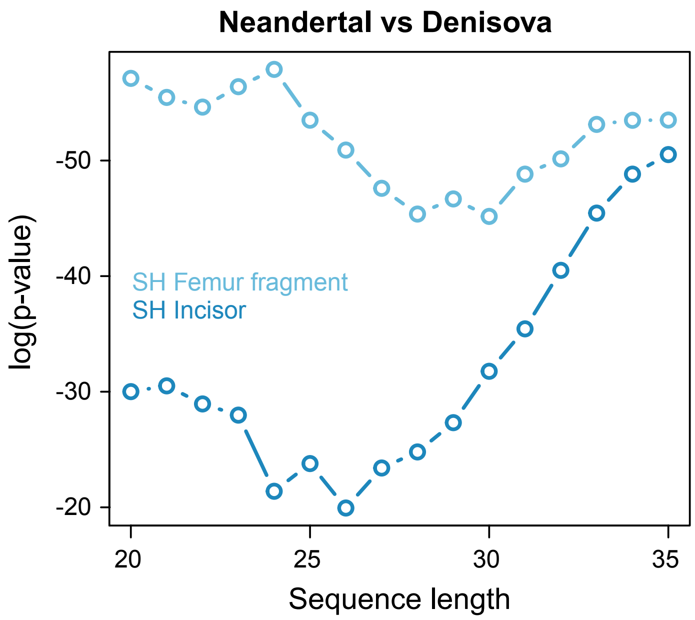

Figure S5. Significance of Neandertal-lineage assignment for different minimum length cutoffs. Fisher exact test p-values in log scale (y-axis) were calculated using read length cutoffs from 20 to 35 bp (x-axis) for the two SH samples (Femur fragment and Incisor) that were previously shown to be significantly closer to Neandertal than Denisova [8]. For the SH Femur fragment the lowest p-value is at 30 bp while for the SH Incisor is at 26 bp, corresponding to an overall percentage of spurious alignments of 2.5% and 15.6% for the two samples, respectively (see Fig. 3).

.

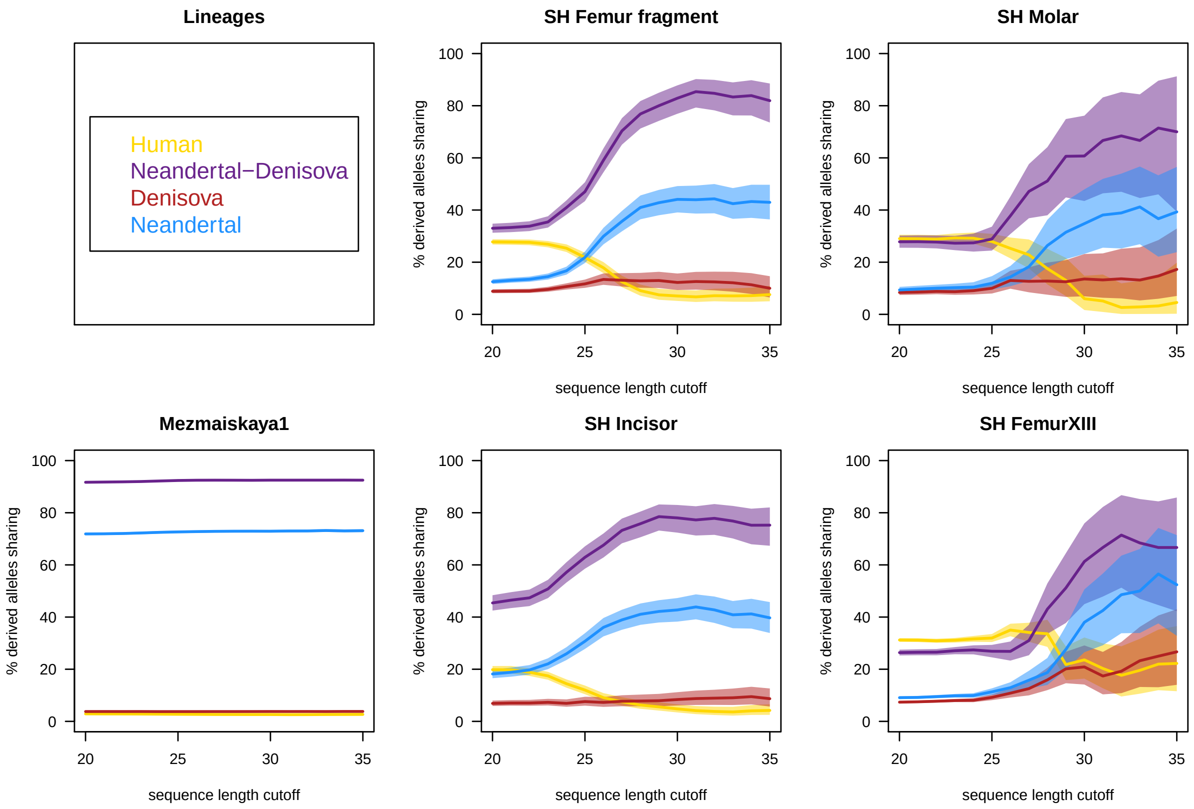

Figure S6. Lineage assignment as a function of sequence length. The percentage of derived allele sharing (y-axis) for four lineages (see legend on the top-left panel) is reported for each sequence length cutoff between 20 and 35 bp. Lines show point estimates and areas indicate 90% binomial confidence intervals.

.
